# The “Podiatry Diabetes Model”- an example of an organised approach to the podiatric care of people with diabetes in regional Victoria

**DOI:** 10.1186/1757-1146-4-S1-O36

**Published:** 2011-05-20

**Authors:** Byron Perrin, Marcus Gardner, Susan Kennett

**Affiliations:** 1 La Trobe Rural Health School, La Trobe University, Bendigo, Victoria, 3550, Australia; 2 Musculoskeletal Research Centre, La Trobe University, Bundoora, Victoria, 3086, Australia; 3 Bendigo Health, Bendigo, Victoria, 3550, Australia; 4 Bendigo Community Health Services, Bendigo, Victoria, 3550, Australia

## Background

To ensure an efficient and consistent publically-funded podiatry service to people with diabetes in the Greater Bendigo region of Victoria the “Podiatry Diabetes Model” of care was developed. The model aims to ensure that people with diabetes are seen by a podiatry service that is most appropriate according to their risk of diabetes-related foot complications. The aim of this study was to determine if people with diabetes were actually seen by the most appropriate podiatry service as indicated by the model.

## Methods

A three-month prospective clinical audit of the Podiatry Diabetes Model was undertaken. Primary variables of interest were the podiatry service where the patients were seen and the patients’ UT Texas risk classification category. For data analysis, the podiatry services were pooled into “community”, “subacute” and “acute” categories. The eight UT Texas risk categories were pooled into “no neuropathy”, “neuropathy”, “history of diabetes-related pathology” and “active diabetes-related pathology” categories. Three separate chi-squared analyses for each service category were undertaken to compare the expected number of patients seen according to foot-health risk as predicted by the model with what was actually observed.

## Results

Five hundred and seventy-six people with diabetes were seen in the three-month period (community: 493; subacute: 67; acute: 16) for 919 contacts (community: 634; subacute: 226; acute: 59). There was significantly more contacts per patient in the subacute and acute services than community service (F = 79.4, p<0.0005). There was no significant difference between the proportion of patients expected by the model to be seen by each podiatry service according to risk status with what was actually observed (community: Χ^2^= 3.3, p= 0.4; subacute: Χ^2^= 8.0, p= 0.05; acute: Χ^2^= 6.6, p= 0.09).

## Conclusions

People with diabetes seen within the Podiatry Diabetes Model are being seen by the most appropriate podiatry service available according to their risk of future diabetes-related foot morbidity. The model is an example of excellent cross-organisation collaboration and could be implemented in other areas of Australia. Future research will investigate whether the long term implementation of the model will influence future incidence rates of ulceration and amputation in the region.

